# Supplementary material for: Ancient DNA reveals a family ossuary and long-distance migration on the Pacific coast before the Inca Empire
Source: Nat Commun. 2026 May 22;17:4222. doi: 10.1038/s41467-026-72216-y (PMC13197425; doi:10.1038/s41467-026-72216-y)
Supplement: Supplementary file 1 — Supplementary Information [file 41467_2026_72216_MOESM1_ESM.pdf]

Supporting Information

Ancient DNA reveals a family ossuary and long-distance migration on the Pacific coast before the Inca Empire

Jacob L. Bongers, Jordan A. Dalton, Erik J. Marsh, Juliana Gómez Mejía, Joshua R. Robinson, Jo Osborn, Emily B. P. Milton, Alexis Rodriguez Yabar, Irving Aragonez Sarmiento, Noemi Oncebay Pizarro, Kalina Kassadjikova, and Lars Fehren-Schmitz

This file includes:

Supplementary Figure 1 | Complex N1 at Las Huacas and Room A2, which was used for the burial of at least 76 individuals.
Supplementary Figure 2 | Conditional heterozygosity calculated within ancient populations where n > 1.

Supplementary Figure 3 | ROH (runs of homozygosity) blocks identified in ancient Andean individuals with > 400,000 SNPs.

Supplementary Figure 4 | Plot of F4-statistics of the type f4(Mbuti.DG, AmericanPopulation; Coast-X, Coast-Y) where "Coast" is either of the three coastal populations along the Peruvian Pacific coast, and "AmericanPopulation" is an ancient or modern-day reference source from the Central Andes, Amazonia, or broader America.

Supplementary Figure 5 | Plot of qpWave p-values for each model testing.

Supplementary Figure 6 | Middle valley cemeteries sampled for aDNA analyses.

Supplementary Figure 7 | Histogram of the very strong ΔR variation.

Supplementary Figure 8 | Birth and death date probability curves for all individuals.

Supplementary Figure 9 | Principal starting and ending boundaries from Bayesian models.

Supplementary Figures

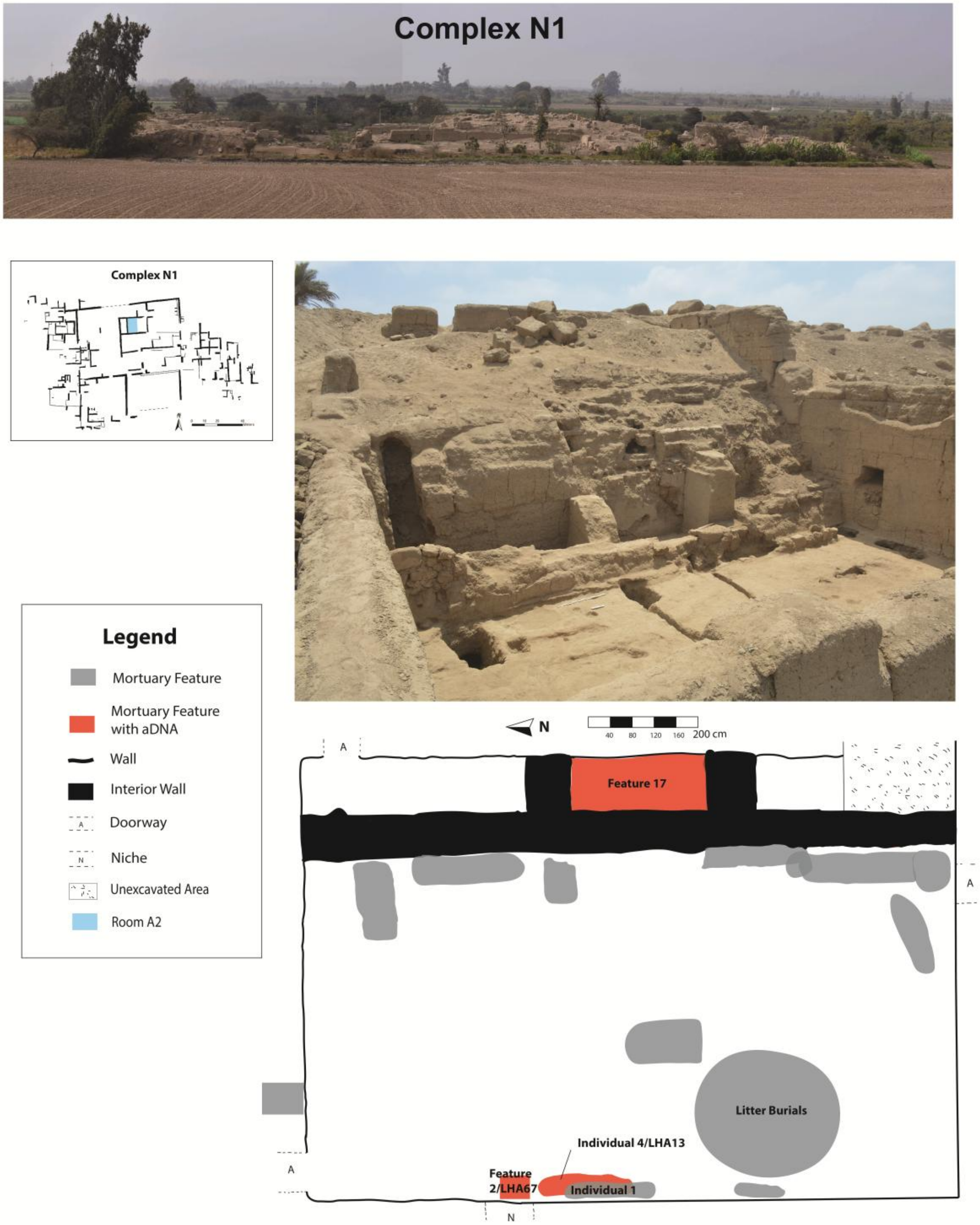

Supplementary Figure 1 | Complex N1 at Las Huacas and Room A2, which was used for the burial of at least 76 individuals. Mortuary features in the room are indicated by grey and red outlines, these features included subterranean tombs, extended individual burials, open-air features, a large communal ossuary (Feature 17), and litter burials.

Features highlighted in red contained individuals that aDNA could be successfully recovered from.

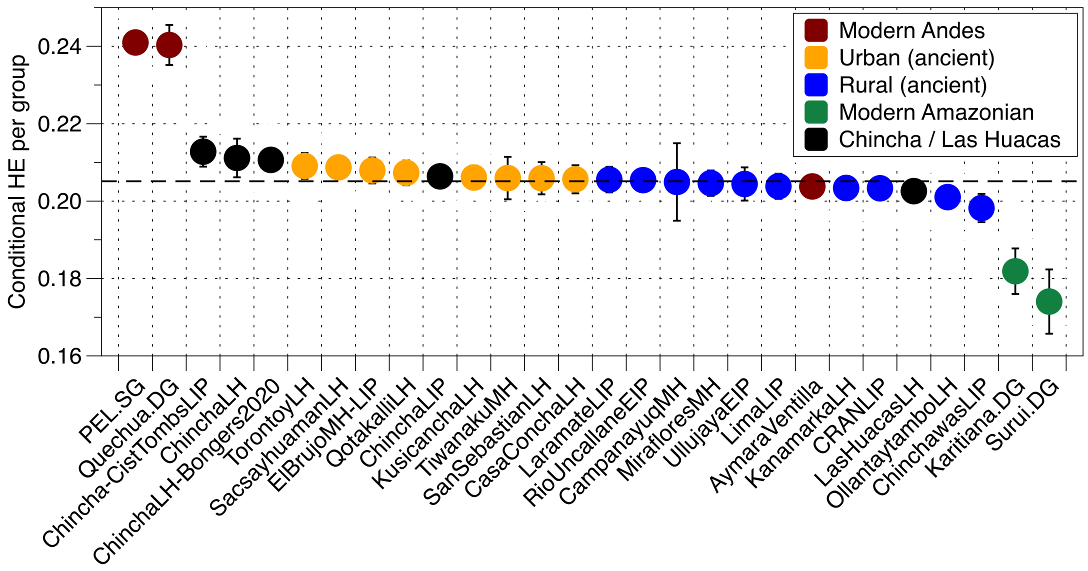

Supplementary Figure 2 | Conditional heterozygosity calculated within ancient populations where n > 1. Thick and narrow error bars represent ± 2 and 3 s.e., respectively. The Chincha Valley groups reported here and from Bongers et al.[18] are plotted in black.

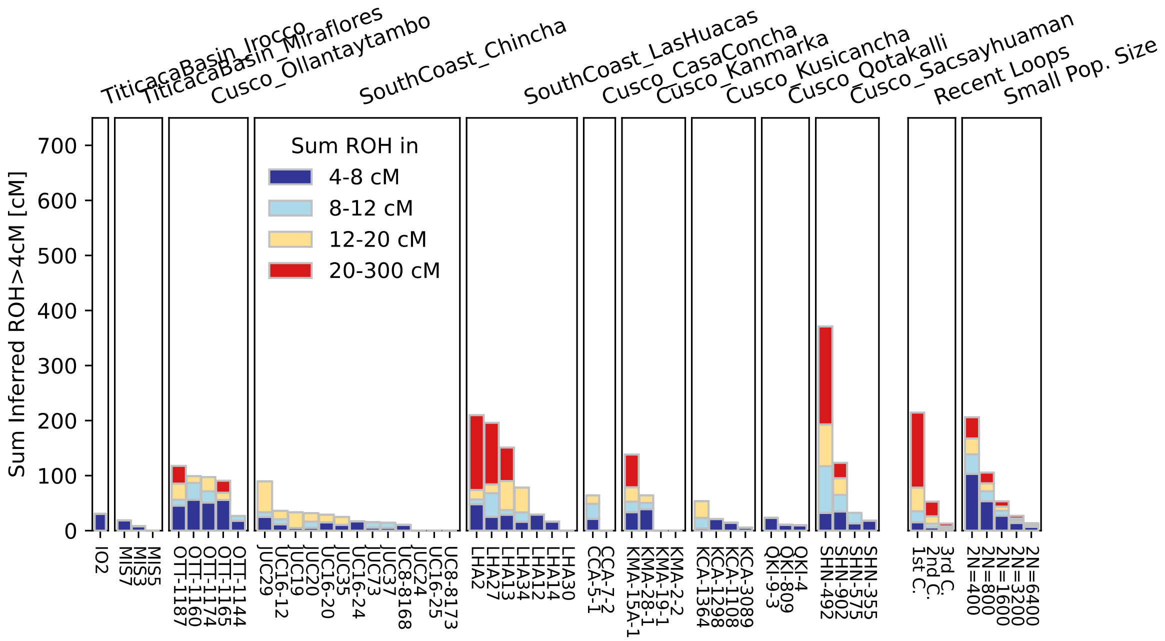

Supplementary Figure 3 | ROH (runs of homozygosity) blocks identified in ancient Andean individuals with > 400,000 SNPs. The two right panels are results from the software's internal standards predicting the distribution of ROH for close paternal relatedness and a small effective population size.

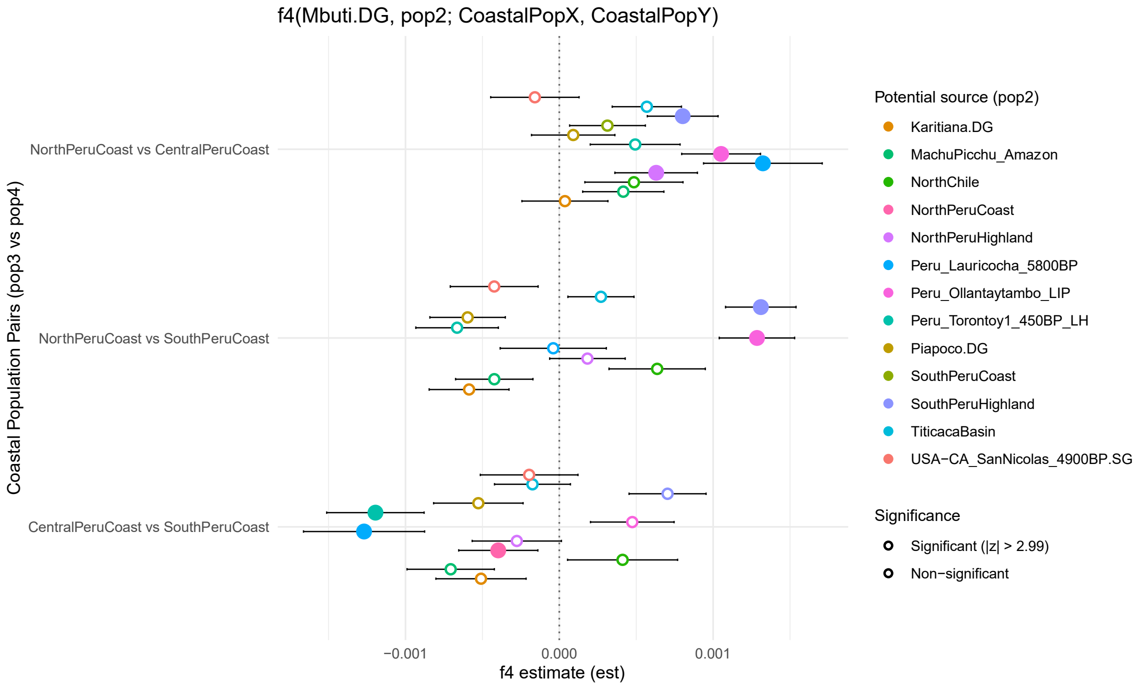

Supplementary Figure 4 | Plot of F4-statistics of the type f4(Mbuti.DG, AmericanPopulation; Coast-X, Coast-Y) where "Coast" is either of the three coastal populations along the Peruvian Pacific coast, and "AmericanPopulation" is an ancient or modern-day reference source from the Central Andes, Amazonia, or broader America. Significant tests (Z = </= -3 or >/= 3) are expressed in filled circles, while non-significant tests are unfilled. The test reveals the genomic structure along the Peruvian coast, indicating differential allele sharing patterns with non-coastal populations.

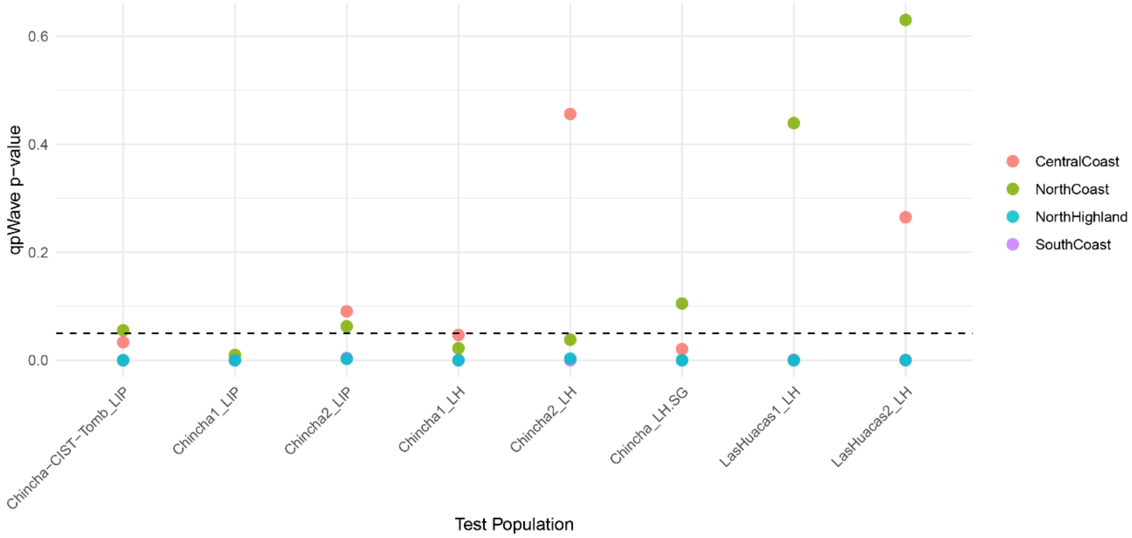

Supplementary Figure 5 | Plot of qpWave p-values for each model testing. P-values above 0.05 indicate that the tested population (x-axis) can be modeled as being consistent with 1-wave of ancestry with the respective coastal reference population (color code see legend), relative to a set of outgroups.

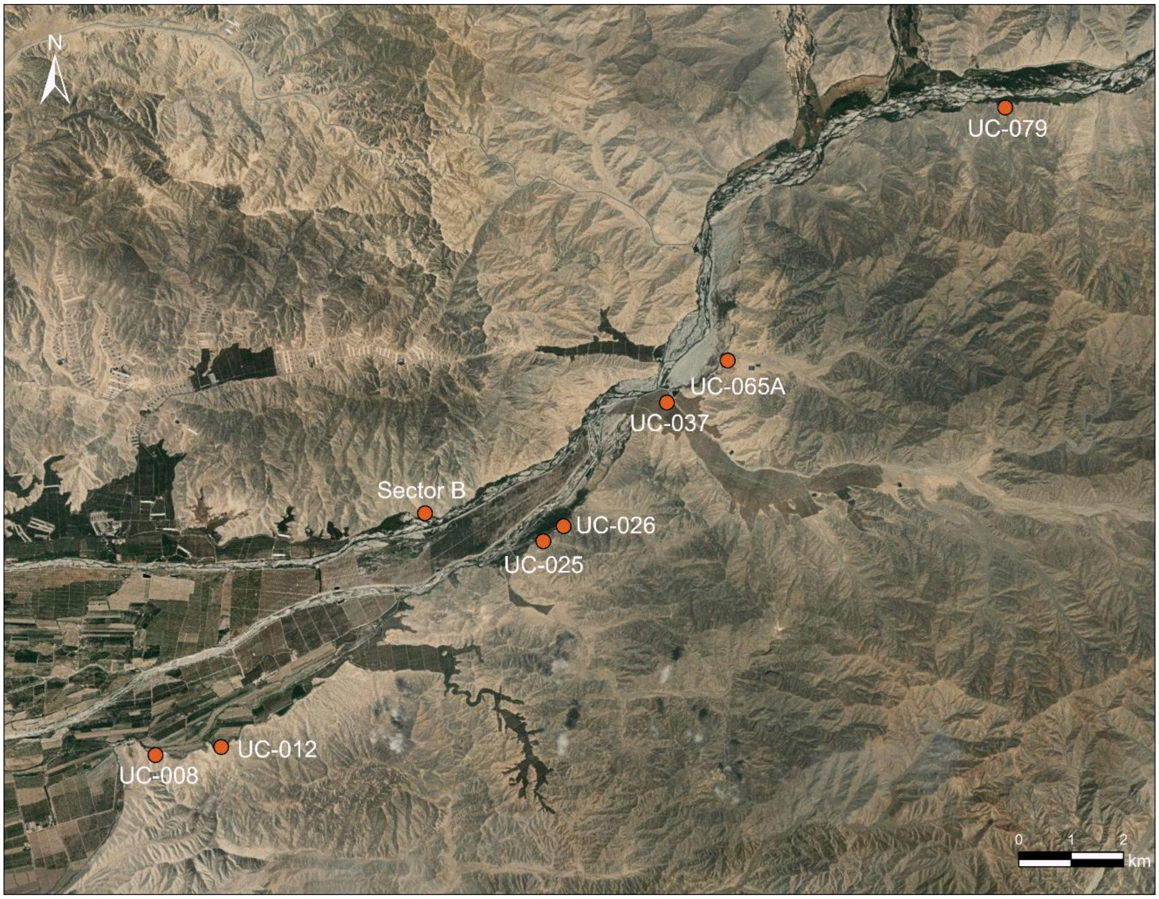

Supplementary Figure 6 | Middle valley cemeteries sampled for aDNA analyses. UC-008 and UC-012 were sampled for Bongers et al.[18]. The other cemeteries were sampled for this paper. The basemap was obtained from the World Imagery dataset (https://www.arcgis.com/home/item.html?id=10df2279f9684e4a9f6a7f08febac2a9) and created with ArcGIS Pro v3.6.2. Sources: Vantor.

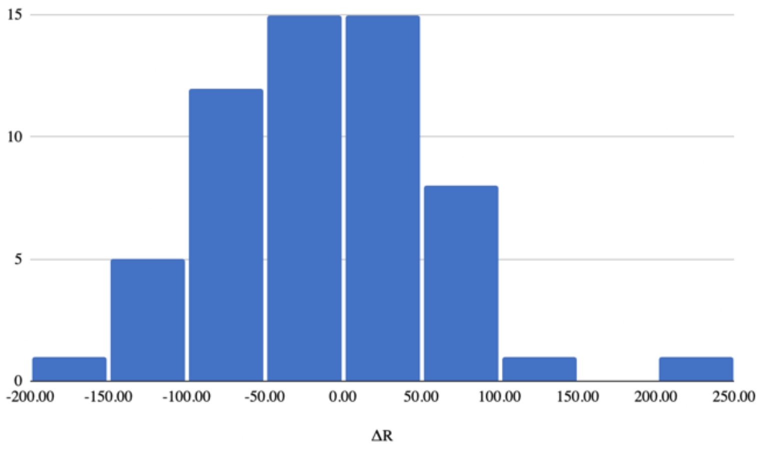

Supplementary Figure 7 | Histogram of the very strong ΔR variation. Based on 58 ^{14}C dates from six shells at four locations in central Peru. The shells were collected pre-bomb, AD 1908–1948[29,30].

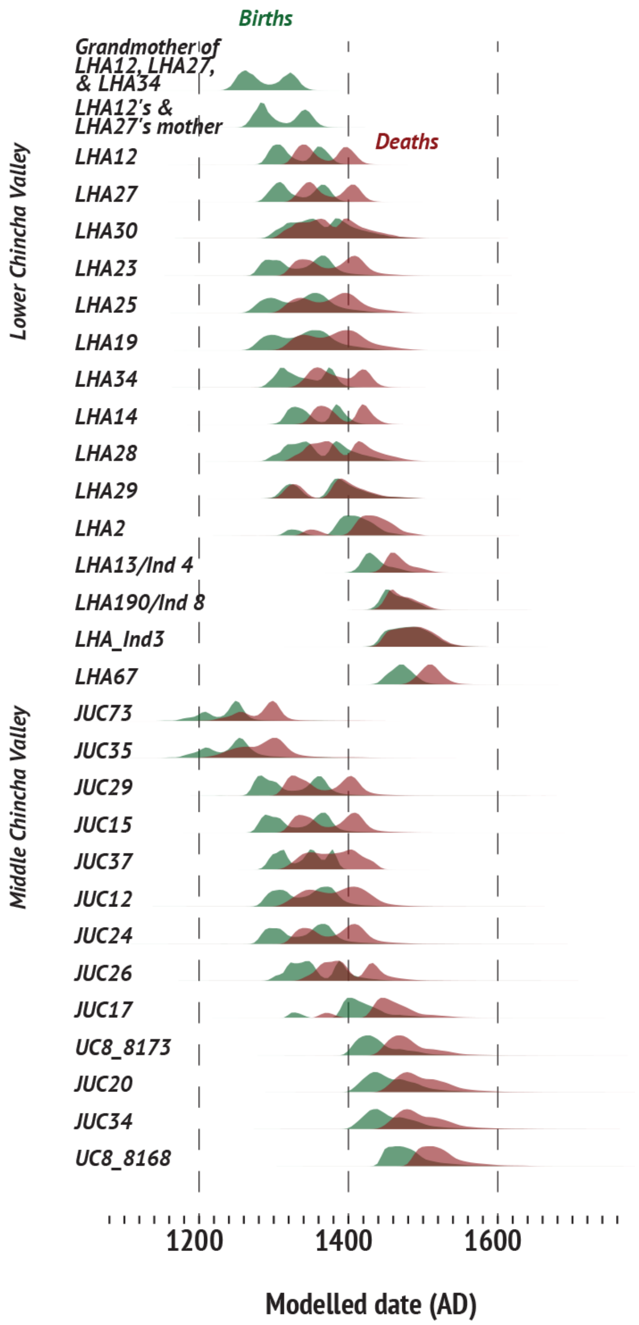

Supplementary Figure 8 | Birth and death date probability curves for all individuals. Based on data in Supplementary Data 1, Table 1b.

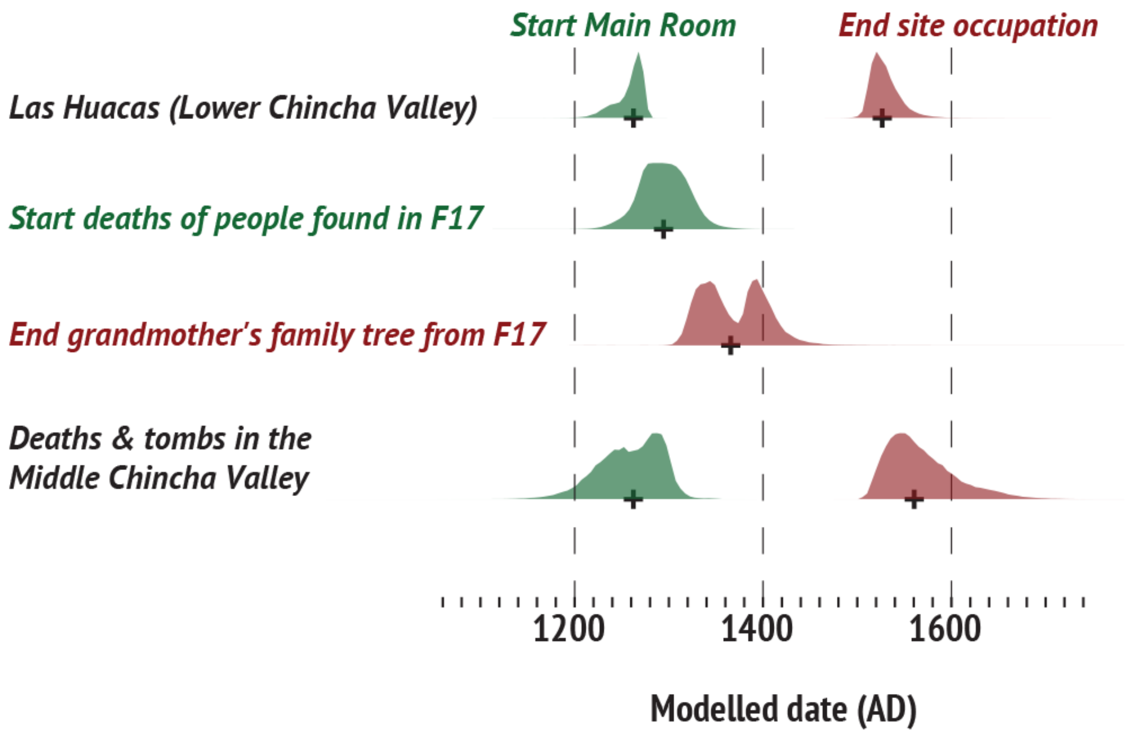

Supplementary Figure 9 | Principal starting and ending boundaries from Bayesian models. Based on data in Supplementary Data 1, Table 1b.
