# Supplementary material for: Ancient DNA reveals a family ossuary and long-distance migration on the Pacific coast before the Inca Empire
Source: Nat Commun. 2026 May 22;17:4222. doi: 10.1038/s41467-026-72216-y (PMC13197425; doi:10.1038/s41467-026-72216-y)
Supplement: Supplementary file 2 — Descriptions of Additional Supplementary Files [file 41467_2026_72216_MOESM2_ESM.pdf]

Description of Additional Supplementary Files

Supplementary Data 1 | A dataset that describes contextual information for the samples and calibration parameters for the radiocarbon data.

Supplementary Data 2 | A dataset that combines all Sequencing Statistics and Population Genetic Analyses on different tabs.

Supplementary Data 3 | A dataset that includes the raw MixSIAR inputs.
