# Supplementary material for: Ancient DNA reveals a family ossuary and long-distance migration on the Pacific coast before the Inca Empire
Source: Nat Commun. 2026 May 22;17:4222. doi: 10.1038/s41467-026-72216-y (PMC13197425; doi:10.1038/s41467-026-72216-y)
Supplement: Supplementary file 6 — Reporting Summary [file 41467_2026_72216_MOESM6_ESM.pdf]

nature portfolio

Corresponding author(s): Jacob L. Bongers, Jordan A. Dalton, Lars Fehren-Schmitz

Last updated by author(s): Mar 18, 2026

Reporting Summary

Nature Portfolio wishes to improve the reproducibility of the work that we publish. This form provides structure for consistency and transparency in reporting. For further information on Nature Portfolio policies, see our Editorial Policies and the Editorial Policy Checklist.

Please do not complete any field with "not applicable" or n/a. Refer to the help text for what text to use if an item is not relevant to your study.
For final submission: please carefully check your responses for accuracy; you will not be able to make changes later.

Statistics

For all statistical analyses, confirm that the following items are present in the figure legend, table legend, main text, or Methods section.

n/a	Confirmed	
☐	☒	The exact sample size (*n*) for each experimental group/condition, given as a discrete number and unit of measurement
☐	☒	A statement on whether measurements were taken from distinct samples or whether the same sample was measured repeatedly
☐	☒	The statistical test(s) used AND whether they are one- or two-sided<br>*Only common tests should be described solely by name; describe more complex techniques in the Methods section.*
☒	☐	A description of all covariates tested
☐	☒	A description of any assumptions or corrections, such as tests of normality and adjustment for multiple comparisons
☐	☒	A full description of the statistical parameters including central tendency (e.g. means) or other basic estimates (e.g. regression coefficient) AND variation (e.g. standard deviation) or associated estimates of uncertainty (e.g. confidence intervals)
☐	☒	For null hypothesis testing, the test statistic (e.g. *F*, *t*, *r*) with confidence intervals, effect sizes, degrees of freedom and *P* value noted<br>*Give P values as exact values whenever suitable.*
☐	☒	For Bayesian analysis, information on the choice of priors and Markov chain Monte Carlo settings
☐	☒	For hierarchical and complex designs, identification of the appropriate level for tests and full reporting of outcomes
☒	☐	Estimates of effect sizes (e.g. Cohen's *d*, Pearson's *r*), indicating how they were calculated

Our web collection on statistics for biologists contains articles on many of the points above.

Software and code

Policy information about availability of computer code

Data collection	Sequencing and isotopic measurements were generated using standard laboratory instrumentation (mass spectrometry, radiocarbon dating facilities, and Illumina sequencing platforms). No custom code was used during data collection.
Data analysis	Data analysis was conducted using a combination of open-source software and custom scripts. MixSIAR v3.l.13 (R package) was used for Bayesian isotopic mixing models. OxCal 4.4 was employed for Bayesian radiocarbon calibration and chronological modeling. Kernel Density Estimation methods in R were applied to summarize chronological distributions. Ancient DNA data were processed using standard bioinformatics pipelines for read mapping, contamination assessment, ROH analysis, f4 statistics, and qpWave (full details in Supplementary Data 2). In addition, a custom R script ("Mix-Cal-Lot") was developed to integrate MixSIAR dietary outputs into OxCal calibrations. The custom Mix-Cal-Lot script to translate estimated marine resource consumption output from MixSIAR to OxCal and the full OxCal script used for this study are available on Zenodo at DOI: https://doi.org/10.5281/zenodo.17917426 and in a .zip file attachment with this manuscript.

For manuscripts utilizing custom algorithms or software that are central to the research but not yet described in published literature, software must be made available to editors and reviewers. We strongly encourage code deposition in a community repository (e.g. GitHub). See the Nature Portfolio guidelines for submitting code & software for further information.

Data

Policy information about availability of data

All manuscripts must include a data availability statement. This statement should provide the following information, where applicable:

- Accession codes, unique identifiers, or web links for publicly available datasets
- A description of any restrictions on data availability
- For clinical datasets or third party data, please ensure that the statement adheres to our policy

All data needed to evaluate the conclusions in the paper are present in the paper and/or the Supplementary Information. Aligned sequencing reads for all individuals reported in this study are available from European Nucleotide Archive (ENA), accession no: PRJEB98110. Human remains and associated materials analyzed in this study are curated in Peru under the authority of the Peruvian Ministry of Culture. Specimens from the Chincha Valley (middle valley and Las Huacas) are housed in local archaeological repositories, such as the lea Regional Museum (museoregionaldeica@cultura.gob.pe), and remain under the care of the Peruvian Ministry of Culture.

Research involving human participants, their data, or biological material

Policy information about studies with human participants or human data. See also policy information about sex, gender (identity/presentation), and sexual orientation and race, ethnicity and racism.

Reporting on sex and gender	N/A
Reporting on race, ethnicity, or other socially relevant groupings	N/A
Population characteristics	N/A
Recruitment	N/A
Ethics oversight	N/A

Note that full information on the approval of the study protocol must also be provided in the manuscript.

Field-specific reporting

Please select the one below that is the best fit for your research. If you are not sure, read the appropriate sections before making your selection.

☐ Life sciences ☒ Behavioural & social sciences ☐ Ecological, evolutionary & environmental sciences

For a reference copy of the document with all sections, see nature.com/documents/nr-reporting-summary-flat.pdf

Behavioural & social sciences study design

All studies must disclose on these points even when the disclosure is negative.

Study description	This is a quantitative, cross-sectional study integrating archaeological, isotopic, radiocarbon, and ancient DNA datasets to investigate kinship, mobility, and dietary practices in pre-Inca Pacific coastal Peru. The study analyzed human skeletal remains from archaeological contexts (ossuaries, tombs, and cist graves) alongside comparative faunal and botanical samples. Quantitative methods included Bayesian isotopic mixing models, Bayesian chronological modeling, and population genetic analyses to reconstruct diet, ancestry, and migration histories.
Research sample	The research sample consisted of 21 archaeologically recovered human individuals from the Chincha Valley, Peru, dating to the Late Intermediate Period through the Inca Empire (ca. AD 1200–1650). Individuals derive from distinct mortuary contexts, including a communal ossuary, tombs, and cist graves. Demographic information such as age-at-death and sex estimation was obtained from osteological analysis when preservation permitted. Comparative datasets included archaeobotanical (C_3 and C_4 plants, maize), faunal (marine and terrestrial), and published isotopic reference materials. The sample is representative of individuals preserved in these contexts but is not intended to represent the entire valley-wide population.
Sampling strategy	Individuals were selected for analysis based on archaeological recovery and preservation quality sufficient for ancient DNA extraction, isotopic analysis, and radiocarbon dating. No formal sample-size calculation was performed; rather, all individuals with adequate preservation and contextual information were included. This yielded a sample of 21 individuals from multiple mortuary contexts in the Chincha Valley. This strategy is standard in ancient DNA analysis and archaeological science, where sample sizes are determined by material availability and preservation constraints rather than statistical power calculations.
Data collection	Data were generated from archaeologically recovered human remains and comparative materials. Ancient DNA was extracted and sequenced using established palaeogenomic protocols in dedicated cleanroom facilities, with sequencing performed on Illumina platforms. Isotopic data ($\delta^{13}C$ and $\delta^{15}N$) were obtained from ultrafiltered collagen using a Fisons NA1500NC elemental analyzer/

	Finnigan Delta Plus isotope ratio mass spectrometer. Radiocarbon dating was performed at the Keck Carbon Cycle AMS facility (University of California, Irvine) following standard pretreatment and quality-control procedures. Contextual archaeological data were derived from excavation and site documentation in the Chincha Valley. All laboratory analyses were conducted by trained specialists following established protocols.
Timing	Archaeological fieldwork and sample recovery in the Chincha Valley were carried out between 2012 and 2018 (Bongers 2019; Dalton 2020, as cited in the manuscript and Supplementary Information). Radiocarbon dating and isotopic analyses were conducted at the Keck Carbon Cycle AMS facility (UC Irvine) between 2018 and 2021, and ancient DNA extraction, sequencing, and genomic analyses were performed at the UCSC Paleogenomics Laboratory between 2019 and 2022.
Data exclusions	Four individuals (JUC27, JUC34, LHA23, LHA28) exhibited contamination rates above the acceptable threshold and were excluded from further genomic analyses. For radiocarbon dating, potential outliers were not removed but formally modeled. For example, a maize sample (OS-149178) had a low agreement index (49.8%), but it was retained and incorporated into the model, as stratigraphic priors and overall model agreement supported its inclusion. This ensured that the dataset remained as complete as possible while accounting for uncertainty.
Non-participation	This study analyzed archaeological human remains; therefore, there were no living participants and no instances of non-participation or dropout.
Randomization	Randomization was not applicable. All available archaeological individuals with sufficient preservation for ancient DNA, isotopic, and radiocarbon analyses were included. Grouping was determined by mortuary context and preservation, not by experimental assignment.

Reporting for specific materials, systems and methods

We require information from authors about some types of materials, experimental systems and methods used in many studies. Here, indicate whether each material, system or method listed is relevant to your study. If you are not sure if a list item applies to your research, read the appropriate section before selecting a response.

Materials & experimental systems

n/a	Involved in the study	
☒	☐	Antibodies
☒	☐	Eukaryotic cell lines
☐	☒	Palaeontology and archaeology
☒	☐	Animals and other organisms
☒	☐	Clinical data
☒	☐	Dual use research of concern
☒	☐	Plants

Methods

n/a	Involved in the study	
☒	☐	ChIP-seq
☒	☐	Flow cytometry
☒	☐	MRI-based neuroimaging

Palaeontology and Archaeology

Specimen provenance	All human remains analyzed in this study were recovered through archaeological fieldwork in two study areas of the Chincha Valley, Peru: the middle valley and the site of Las Huacas. Fieldwork, export, and laboratory analyses were conducted under permits issued by the Peruvian Ministry of Culture. For the middle valley, permits were granted in 2013 (206-2013-DGPC-VMPCIC/MC), 2015 (218-2015-DGPA-VMPCIC/MC), 2016 (107-2016-VMPCIC-MC), 2017 (145-2017-DGPA-VMPCIC/MC), and 2018 (148-2018-DGPA-VMPCIC/MC). For Las Huacas, permits were granted in 2017 (001379-2017/DGPA/VMPCIC/MC) and 2019 (035-2019-VMPCIC-MC, 101-2019-VMPCIC-MC).
Specimen deposition	Human remains and associated materials analyzed in this study are curated in Peru under the authority of the Peruvian Ministry of Culture. Specimens from the Chincha Valley (middle valley and Las Huacas) are housed in local archaeological repositories, such as the Ica Regional Museum, and remain under the care of the Peruvian Ministry of Culture.
Dating methods	A total of 43 radiocarbon determinations were modeled in this study. Samples were obtained directly from archaeologically excavated human remains and associated botanical materials (primarily maize). Following fieldwork in the Chincha Valley, Peru, samples were stored in secure local repositories under the authority of the Peruvian Ministry of Culture until export was authorized. Upon export, subsamples were transferred under controlled conditions to the Keck Carbon Cycle AMS facility (University of California, Irvine) for radiocarbon measurement. Laboratory pretreatment followed standard acid–base–acid protocols, with ultrafiltration applied where appropriate. Radiocarbon ages were calibrated in OxCal v4.4. Quality assurance included assessment of collagen integrity (C:N ratios, %C, %N, and collagen yields) and application of accepted thresholds for inclusion. Full laboratory codes, pretreatment details, and model outputs are provided in the Supplementary Information.

☒ Tick this box to confirm that the raw and calibrated dates are available in the paper or in Supplementary Information.

Ethics oversight	All archaeological fieldwork, sampling, export, and laboratory analyses were conducted with the approval and oversight of the Peruvian Ministry of Culture. Permits for excavation, sampling, and export were issued by the Ministry (see permit numbers listed above).

Note that full information on the approval of the study protocol must also be provided in the manuscript.

Plants

Seed stocks	N/A
Novel plant genotypes	N/A
Authentication	N/A

This checklist template is licensed under a Creative Commons Attribution 4.0 International License, which permits use, sharing, adaptation, distribution and reproduction in any medium or format, as long as you give appropriate credit to the original author(s) and the source, provide a link to the Creative Commons license, and indicate if changes were made. The images or other third party material in this article are included in the article's Creative Commons license, unless indicated otherwise in a credit line to the material. If material is not included in the article's Creative Commons license and your intended use is not permitted by statutory regulation or exceeds the permitted use, you will need to obtain permission directly from the copyright holder. To view a copy of this license, visit http://creativecommons.org/licenses/by/4.0/
